# Utility of Cardiac Rehabilitation Following Surgical Treatment of Infective Endocarditis

**DOI:** 10.31083/RCM45789

**Published:** 2025-12-23

**Authors:** Sara M. Telles-Langdon, Ali Fatehi Hassanabad

**Affiliations:** ^1^Section of Cardiac Surgery, Department of Cardiac Sciences, Libin Cardiovascular Institute, Cumming School of Medicine, Calgary, AB T2N 2T9, Canada

**Keywords:** infective endocarditis/endocarditis, cardiac rehabilitation, cardiac surgery/thoracic surgery, social determinants of health

## Abstract

Infective endocarditis (IE) is a life-threatening cardiac infection. The incidence of IE is increasing due to complex sociodemographic shifts, including increases in intravenous drug use (IVDU) attributed to opioid epidemics. Cardiac rehabilitation (CR) is a comprehensive form of secondary prevention for heart disease. Current guidelines suggest that CR may be beneficial in the recovery from IE, but supporting evidence is limited. Given the utility of CR in the recovery from other cardiac conditions and the unique characteristics of patients with IE, this narrative review summarizes the existing data on the use of CR following surgical treatment of IE. The existing literature is limited to the CopenHeart_IE_ randomized clinical trial (RCT) and four case reports. Thus, to our knowledge, this represents the first review to focus specifically on CR in the context of IE. The CopenHeart_IE_ RCT found that patients receiving CR showed greater improvements in levels of physical fatigue, general fatigue, maximal power, systolic blood pressure, and some questionnaire scores than the control group. The results of multiple case reports represent unique and extreme cases of IE from which support for the use of CR following IE can be drawn from the relative successes of each patient. Moreover, it is important to consider that the complex social needs of the IE population may require additional psychosocial support, which can be achieved by integrating social workers into the multidisciplinary CR team. While further research is warranted, the existing evidence supports the use of CR as part of the comprehensive recovery from IE.

## 1. Introduction

Infective endocarditis (IE) is a life-threatening cardiac infection that often requires surgical treatment [1, 2]. Despite being relatively rare, IE presents a major public health challenge [1, 2]. As the most severe complication of heart valve disease, surgical treatment of IE is associated with the highest mortality of any valve disease [3]. IE has an increasing annual incidence of up to 12.61 per 100,000 people, and has a ~25% mortality rate, accounting for 77,840 deaths worldwide in 2021 [1, 2, 4]. Perhaps the most devastating statistic is that the disability adjusted life years (DALYs) attributable to IE were 2,076,413 in 2021, representing an increase globally since 1990 [4]. Therefore, research to optimize recovery and reduce the associated morbidity of IE is of paramount importance.

The primary contributing risk factors for IE include rheumatic heart disease (RHD), cardiac implants such as prosthetic valves and indwelling electronic devices, and intravenous drug use (IVDU) [2]. While the incidence of IE is increasing globally, the relative contribution of each category varies between countries with different socio-demographics and healthcare systems [2]. The incidence of RHD-associated IE is decreased in healthcare systems with more robust management of the underlying rheumatic disease [2]. The incidence of cardiac implant-associated IE is increasing, likely due to the increased use of implantable devices worldwide, as advances are made in medical technology and surgical techniques [2, 5]. While sterile techniques help mitigate the risk, the possibility of infection still exists with every invasive procedure [5]. Additionally, there has been a worldwide increase in antibiotic resistance, which contributes to the mortality associated with IE. This can be mitigated by limiting the prescription of cephalosporins, as demonstrated by the lower incidence of IE in countries with antimicrobial stewardship [2]. IVDU is a complex social determinant of health (SDOH) that is a significant contributor to the incidence of IE, particularly in countries facing opioid epidemics [2, 5].

In the United States, recent trends in IE incidence and mortality have mirrored the increases in IVDU attributed to the opioid epidemic [5]. The states that reported the highest rates of opioid-involved overdose deaths in 2018 matched the states with the highest age-adjusted mortality rates of IE from 2010 to 2019 [5]. Populations that have had notable increases in IE-associated mortality include adults under age 65 and rural populations. Prior to the opioid epidemic, the incidence of IE among young adults was extremely low, but rates of IVDU-associated IE have been increasing since 2010, primarily affecting younger adults previously at low risk of developing IE [5]. The mortality rate of IE has also increased in rural populations despite a decreased mortality rate in urban populations. This discrepancy is likely attributable to disparities in the accessibility of healthcare in rural communities [5].

The overdose rates in the United States continue to climb, with a 30% increase from 2019 to 2020, leading to increased hospitalizations for IVDU-related injuries, including IE [6]. This patient population requires additional consideration of the SDOH that contribute to both the risk of IVDU-related injuries and to patient recovery. When considering the intersectionality of race, gender identity, income, ability, sexual identity, and other SDOH that contribute to health equity, and the social stigma surrounding IVDU, a significant portion of the IE population may need additional social supports and considerations for a successful recovery [6]. Many hospitals have implemented multidisciplinary teams including physicians, social workers, and peer support workers to help address the unique challenges faced by patients with a history of IVDU and to improve health equity [6]. While we have drawn from studies conducted in the United States, similar opioid epidemics, IE trends, socioeconomics, and health equity concerns are observed in other countries.

The 2020 American College of Cardiology (ACC) and American Heart Association (AHA) guidelines for the management of valvular heart disease offer recommendations about antibiotic use, medical therapy, and surgical intervention for IE, but do not make any recommendations for long-term recovery [7]. The 2023 European Society of Cardiology (ESC) guidelines for the management of endocarditis recommend that “cardiac rehabilitation (CR) including physical exercise training should be considered in clinically stable patients based on an individual assessment.” [1]. The guidelines state that CR has been shown to be safe and feasible in stable patients and that it may be beneficial, that CR should start as early as possible after surgery and can be adapted to isolated lower-limb training post-sternotomy, that earlier initiation of CR improves adherence, and that reducing frailty and building muscle mass should be a priority [1]. All of these recommendations were based on the results of the CopenHeart_IE_ randomized clinical trial (RCT) [8]. Since the results of the RCT were statistically weak, the ESC categorized the recommendation as Class IIa. This classification stipulates that the intervention should be considered because the evidence is in favour of efficacy, but that there is “conflicting evidence and/or a divergence of opinion about the usefulness/efficacy of the given treatment or procedure.” [1].

CR is a comprehensive form of secondary prevention for heart disease that includes patient education, risk factor modification, exercise training, dietary counselling, and psychosocial support [9]. In Canada, CR has been a part of patient care since the 1970s [10]. There are now ~220 CR programs throughout Canada that support more than 50,000 new patients every year [10]. Participation in these programs has been shown to decrease patient morbidity and mortality by improving exercise capacity and lipid profile while reducing inflammation, obesity, and psychological distress [10]. While the evidence shows that CR significantly reduces the morbidity and mortality associated with cardiovascular disease, it is still underutilized, and access to proper CR remains inequitable [9, 10].

Considering the high morbidity and mortality associated with IE, and the utility of CR in the recovery from other cardiac conditions, this narrative review aims to describe the existing data on the utility of CR following surgical treatment of IE. This review also considers the unique socioeconomic characteristics of the IE patient population. The existing literature on CR following surgical treatment of IE is limited to an RCT and case reports. To the extent of our knowledge, this is the first review specific to CR in the context of IE.

## 2. Methods

A search of the PubMed database was completed on December 6, 2024, using the search terms presented in Table 1. Abstracts and keywords of all English language results were reviewed, and articles were included in the initial review based on the presence of both CR and IE or closely related terms. All types of studies and all publication dates were included. Studies on non-IE cardiac conditions, CR for non-IE cardiac conditions, or IE recovery with no CR were excluded. After the initial search, the subsequent searches continued to return subsets of the same relevant articles. Searches of other databases did not return any additional studies that were deemed to be of high rigour. For the analysis, each study was read in detail, and we are reporting a summary and assimilation of the existing research in the context of other similar studies. Due to the small number of available articles, a quality appraisal was not conducted, and all available articles were included in a comprehensive narrative review.

**Table 1.** **Search terms used in the PubMed database**.

Search terms	Search results	Included results
(infective endocarditis) AND (“cardiac rehabilitation”)	32	10
(“infective endocarditis”) AND (“cardiac rehabilitation”)	15	8*
(“endocarditis”) AND (“surgery”) AND (“cardiac rehabilitation”)	20	9*
(“intravenous drug use”) AND (“endocarditis”) AND (“cardiac rehabilitation”)	0	0
(“intravenous drug use”) AND (“surgery”) AND (“cardiac rehabilitation”)	0	0
(“intravenous drug use”) AND (“cardiac rehabilitation”)	0	0
(intravenous drug use) AND (“cardiac rehabilitation”)	16	0

*Articles also returned in earlier search.

Ten articles were found that seemed relevant to the research question based on an initial review. After a more in-depth analysis of those ten articles, it was discovered that only eight articles were specific to IE [8, 11, 12, 13, 14, 15, 16, 17]. The additional two articles were excluded from the analysis because they only included IE as a possible surgical complication or as a study endpoint [18, 19]. While they will not be included in the results, the additional two articles present some supporting evidence that will be included in the discussion [18, 19]. The eight remaining articles were reviewed to compare the results of each study.

## 3. Results

**Fig. 1.**
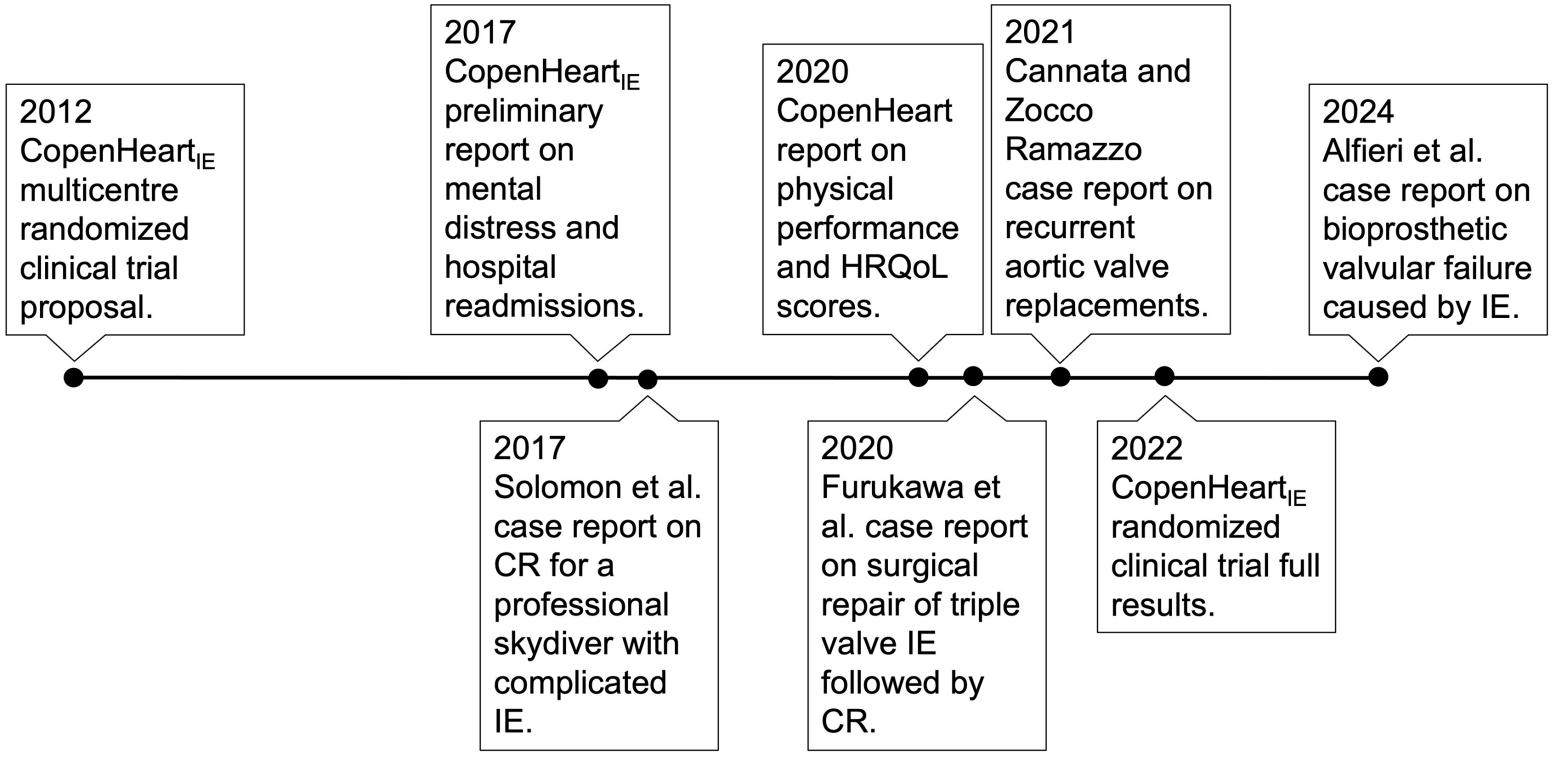
**Literature timeline**. Publication timeline of the existing literature on CR following treatment of IE. CR, cardiac rehabilitation; IE, infective endocarditis; HRQoL, Health-Related Quality of Life.

Of the eight included articles, four articles are at different stages and analyses from the large CopenHeart trials [8, 11, 12, 13]. The other four articles are case reports of unique presentations of IE [14, 15, 16, 17]. Considering the context, limitations, and strengths of each study, the information presented is considered to guide the discussion and recommendations of this narrative review. Fig. 1 provides a timeline of the various trials and studies that have focused on CR in this patient population. A summary of the results is presented in Table 2.

**Table 2.** **Research design and main outcomes of included articles**.

Title	Authors	Research design	Main outcomes
A randomised clinical trial of comprehensive cardiac rehabilitation versus usual care for patients treated for infective endocarditis - the CopenHeart_IE_ trial protocol	Rasmussen *et al*.	Randomized clinical trial protocol	Study protocol designed to compare patient outcomes between comprehensive CR and the usual standard of care following IE.
High readmission rates and mental distress after infective endocarditis - Results from the national population-based CopenHeart_IE_ survey.	Rasmussen *et al*.	Randomized clinical trial	High rates of hospital readmission following IE are associated with low self-reported mental and physical health.
Changes in physical performance and their association with health-related quality of life in a mixed nonischemic cardiac population that participates in rehabilitation.	Tang *et al*.	Randomized clinical trial	Correlations between improved physical performance and health questionnaire scores in nonischemic cardiac populations.
Comprehensive cardiac rehabilitation for patients following infective endocarditis: results of the randomized CopenHeart_IE_ trial.	Rasmussen *et al*.	Randomized clinical trial	CR showed some benefits to physical fatigue, general fatigue, maximum power, systolic blood pressure, and HRQoL scores following IE.
CR for a skydiver after aortic valve replacement for pure aortic regurgitation and resection of the ascending aorta complicated by active infective endocarditis and heart block requiring a pacemaker.	Solomon *et al*.	Case report	Personalized CR was successful for return to professional skydiving after surgical treatment of complicated IE.
A surgical case of triple valve replacement for triple valve endocarditis with multiple vegetations.	Furukawa *et al*.	Case report	CR was successful after surgical treatment of triple valve IE in a patient with multiple severe comorbidities.
Echocardiographic evaluation of paravalvular aortic regurgitation of a patient with recurrent aortic valve replacements.	Cannata and Zocco Ramazzo	Case report	CR was successful in recurrent aortic valve replacements for IE.
There is nothing more invisible than the obvious: A case summary and literature review.	Alfieri *et al*.	Case report	CR was successful following bioprosthetic valvular failure caused by IE.

CR, cardiac rehabilitation; IE, infective endocarditis; HRQoL, Health-Related Quality of Life.

### 3.1 The Conception of a Randomized Clinical Trial

In 2012, Rasmussen *et al*. [11] proposed the CopenHeart_IE_ multicentre RCT as part of the CopenHeart project developing evidence-based rehabilitation for complex cardiac conditions. The study represents the first and only RCT examining the effects of CR following treatment of IE [8, 11, 12, 13]. Based on the evidence that CR is effective for patients with coronary heart disease, heart failure, and recovering from valve replacement surgery, the CopenHeart_IE_ trial was designed to compare patient outcomes between comprehensive CR and the usual standard of care following IE using both quantitative and qualitative outcome measures [11]. The control group patients received standard follow-up visits, including a clinical assessment, vitals, bloodwork, and a transthoracic echocardiogram (TTE). In addition to this standard of care, the experimental group patients completed physical training and psychoeducational consultations starting four weeks after hospital discharge and continuing for twelve weeks. The physical training included three sessions per week consisting of both aerobic and resistance training for a total of 60 minutes per session, for a total of 36 hours [11]. The patient outcomes to be measured included mental health and physical capacity [11].

### 3.2 CopenHeart_IE_: Preliminary Report

The first CopenHeart_IE_ results were published in 2017 [12]. Although the RCT was not yet complete, the investigators released a preliminary report on mental distress and hospital readmission rates following IE. They reported that within the first year following discharge after initial IE treatment, 65% of patients had to be rehospitalized, and 18% died. The 186 rehospitalized patients had a combined total of 483 readmissions, with an average of 2.6 readmissions per patient [12]. The study participants completed the Short Form 36 Health Survey Questionnaire (SF-36), a questionnaire designed to measure quality of life (QoL), shortly after initial hospital discharge. In addition to the high rates of readmission, study participants had significantly lower SF-36 scores compared to background-matched controls and compared to patients recovering from heart valve surgery without IE. Higher rates of rehospitalization seemed to be associated with lower self-reported mental and physical health [12]. Although the outcomes of CR participation were not discussed in this preliminary report, 41% of the study patients participated in CR, and the authors propose that patients could benefit from CR, considering the significant vulnerability to physical deconditioning and psychological distress of the IE population [12].

### 3.3 CopenHeart_IE_: Follow-up Results

Further results of the CopenHeart_IE_ RCT were released in 2020 in combination with results from other studies in the CopenHeart project focusing on CR in nonischemic cardiac populations [13]. The article reported on results from three RCTs conducted simultaneously with a parallel design, specifically focusing on the correlations between physical performance and Health-Related Quality of Life (HRQoL) questionnaire scores [13]. Most of the correlations were categorized as weak or very weak. Increases in maximum power were significantly correlated with four of the five HRQoL scores. Improvements in the six-minute walk test (6MWT) and the sit-to-stand test repetitions were significantly correlated with increased SF-36 scores. Improved sit-to-stand test scores were also significantly correlated with increased HeartQoL scores [13]. Correlations between improved physical performance and increased HRQoL scores were more strongly associated with the physical health dimensions of the HRQoL than with the emotional dimensions [13]. The authors also highlighted that there was variation between age, sex, and each individual heart diagnosis [13], suggesting that personalized CR programs may be more beneficial than generic CR.

### 3.4 CopenHeart_IE_: Final Results

In 2022, Rasmussen *et al*. [8] published the full results of the CopenHeart_IE_ RCT. As previously described, the study compared quantitative and qualitative patient outcomes after IE between a control group receiving standard follow-up care and an experimental group receiving comprehensive CR in addition to the standard follow-up [8, 11]. There was no statistically significant difference between the two groups at the end of the intervention for the primary outcome of mental health as measured by the SF-36. However, the SF-36 mental health scores of the experimental group were significantly lower than the scores of the control group at baseline [8]. Similarly, there was no significant difference between the two groups at baseline or at the end of the intervention for the secondary outcome of physical capacity [8]. Four additional exploratory outcomes showed differences between the two groups. Patients in the experimental group had greater improvements in levels of physical fatigue and general fatigue than the control group. The experimental group also had improved maximal power and systolic blood pressure compared to the control group [8]. Interpretation of this data should be done skeptically, as the study did not reach the target sample size for statistical power. Additionally, the experimental group had only 43% adherence to the physical training, 60% adherence to the psychoeducational consultations, and 28% adherence to both programs. The data may also be confounded because 36% of the control group participated in some form of CR program outside the study that is not part of the standard follow-up care [8].

### 3.5 Supporting Evidence From Case Reports

In 2017, Solomon *et al*. [14] published a case report on successful CR for a professional skydiver with complicated IE. Following an aortic valve replacement with implantation of an aortic tube graft, the patient developed IE and an ascending aortic abscess that was treated using surgical debridement and implantation of a dual-chamber pacemaker. The patient began his recovery at a generic CR program in Brazil, then enrolled in the Baylor Hamilton Heart and Vascular program in the United States for more specific testing and exercise training [14]. On initial assessment, the patient was weak and debilitated from his extensive hospitalization. Skydiving-specific cardiovascular and strength tests were performed at the beginning and end of the CR program [14]. The patient underwent customized training to simulate skydiving with close monitoring of symptoms and cardiac function [14]. He showed a 22.5% improvement in muscular strength and an improved metabolic stress test result. After completing the personalized CR program, the patient successfully returned to professional skydiving [14]. While this report followed an extreme case of a professional athlete, the results still provide some insight into the potential success of individualized CR programs.

In 2020, Furukawa *et al*. [15] published a case report on the surgical repair of triple valve IE followed by CR. The patient presented with a left temporal lobe cerebral infarction, a malignant ileocecal tumor, multiple mobile vegetations on the aortic, mitral, and tricuspid valves, and regurgitation in all three valves [15]. The patient underwent open triple valve replacement surgery under cardiopulmonary bypass [15]. Once the patient was transferred from the intensive care unit to the general ward, he underwent CR, recovered, and was discharged from the hospital approximately three months after surgery. The patient died of multiple organ failure nine months after the triple valve replacement with no signs of any infection-related postoperative complications [15]. While this report described a very severe form of IE in a patient with multiple comorbidities, the surgical treatment and CR appeared to be successful [15].

In 2021, Cannata and Zocco Ramazzo [16] published a case report on a patient requiring recurrent aortic valve replacements. In 2004, the patient presented with a large mobile vegetation on the aortic valve with severe regurgitation [16]. The patient then underwent a valve replacement with a biological prosthesis. In 2019, the patient presented with IE of the bioprosthesis [16]. This presentation was treated with a second bioprosthetic valve replacement. Later in 2019, the patient presented with massive paravalvular regurgitation (PVR) due to a large abscess cavity. This presentation was treated with a third aortic valve replacement with a mechanical valve and aortic root reinforcement. The patient participated in CR after each surgery [16]. The patient remained asymptomatic at their one-year follow-up appointment for the third valve replacement [16].

Most recently, in 2024, Alfieri *et al*. [17] published a case report on bioprosthetic valvular failure caused by IE. The patient presented three months after an aortic valve replacement with severe aortic PVR due to prosthesis detachment and pseudoaneurysm development. The patient also had multiple comorbidities, including arterial hypertension, peripheral artery disease, chronic obstructive pulmonary disease, and diabetes mellitus [17]. The patient underwent a second aortic valve replacement with pseudoaneurysm repair. The patient was then referred to a CR program where he had a complete recovery [17].

## 4. Discussion

### 4.1 Infective Endocarditis Study Outcomes

Considering the CopenHeart_IE_ RCT, there were very limited differences between the control group receiving the standard of care and the experimental group receiving comprehensive CR [8, 11, 12, 13]. However, the CR did show some benefits to physical and general fatigue, maximum power, systolic blood pressure, and the physical health dimensions of HRQoL scores [8, 13]. The inconclusive nature of the CopenHeart_IE_ results could be explained by baseline differences between the two study groups, a lack of statistical power, and poor adherence to the CR and the standard of care. The experimental group had lower fitness and mental health scores at the start of the intervention than the control group, so while there were very few differences between the groups at the end of the intervention, the relative within-group improvement was greater in the experimental group than the control group [8]. In order to reach statistical power, the study required a minimum of 150 participants. Despite extending the recruitment period to 5 years, which was longer than originally planned, the trial concluded with only 117 participants [8, 11]. The insufficient sample size means that there may have been true differences between the two groups that are not represented as significant in the statistical analysis, but could become apparent with sufficient data. Perhaps the most likely contributor to the lack of significant differences between the two groups was poor adherence to the study protocol in both the control group and the experimental group. The standard of care for IE recovery used in the study did not include any form of CR. However, 36% of the participants attended CR programs outside of the study [8]. This elective CR participation would shift the control group closer to the experimental group.

Similarly, only 28% of the participants in the experimental group adhered to both the physical training and psychoeducational consultations required for the CR program. The two components individually had better adherence [8], but the low CR participation would shift the experimental group closer to the control group. If we consider the most extreme hypothetical that 28% of the experimental group and 36% of the control group fully participated in similar CR programs, then it is unsurprising that any potential benefits of CR were not represented in the between-group analysis. Additionally, the rates of CR participation in both groups prompt the consideration that only 30–40% of IE patients will participate in CR, regardless of recommendations from guidelines or their care team. Subsequently, it is worth considering what participation barriers exist for the subset of patients who are unable to complete a CR program.

Despite the lack of conclusive results, the CopenHeart_IE_ RCT still supports the notion that some form of CR is likely beneficial in the recovery from IE. The preliminary report on mental distress and hospital readmissions is of particular importance in the IE population due to the significant vulnerability to physical deconditioning and psychological distress [12]. Since improving both physical fitness and mental health are the primary goals of CR [9], it is logical to conclude that participation in effective CR may improve patient outcomes and decrease the likelihood of rehospitalization. The combined CopenHeart report on patients recovering from nonischemic heart disease highlighted that there was variation in the response to CR between age, sex, and each individual heart diagnosis [13]. This suggests that a one-size-fits-all approach may not be effective for certain patient populations and that personalized CR programs may be more beneficial. This idea is particularly evident in some of the existing case reports.

### 4.2 Lessons Learned From Case Reports

While each patient discussed in the four case reports represents a unique and extreme case, support for CR following IE can be drawn from the relative successes of each patient. In the case reported by Alfieri *et al*. [17], the patient developed IE that led to a paravalvular pseudoaneurysm, the most lethal type of bioprosthetic valvular failure. After the second valve replacement and surgical repair of the pseudoaneurysm, CR was an important part of the patient’s recovery. Despite facing a severe complication after the initial surgery, the patient was able to safely participate in CR and recovered fully [17]. In the case reported by Cannata and Zocco Ramazzo [16], the patient underwent three aortic valve replacements for recurrent IE and participated in CR after each surgery. Considering the ~25% mortality rate of IE, recovery from recurrent IE requiring multiple surgeries is impressive [1, 2]. The patient continued to attend CR even after the third surgery, presumably because he found some amount of benefit from CR after the first and second surgeries. In the case reported by Furukawa *et al*. [15], the patient underwent triple valve replacement for IE of three valves. The patient also had a cerebral infarction and a malignant ileocecal tumor. Despite having hemiplegia and muscle weakness from the cerebral infarction, the patient was able to participate in CR. The patient died of multiple organ failure, likely due to malignancy, nine months after the cardiac surgery. This case demonstrates that even in the sickest patients with multiple comorbidities, CR can be a safe component of IE recovery.

In the case reported by Solomon *et al*. [14], the patient underwent an initial aortic valve replacement with implantation of an aortic tube graft, then developed IE requiring a second surgery for debridement of the infection and implantation of a pacemaker. After surgery, the patient sought out a CR program that would provide specific testing and exercise training with the goal of returning to professional skydiving [14]. This case provides the most detailed report available of a customized CR program following IE. This case also demonstrates the potential positive outcomes of CR, as the patient was able to recover from a weak and debilitated state to a fitness level far superior to that of many healthy people with no history of cardiac issues. While returning to competition as a professional athlete is not the goal of most IE patients, this case shows the feasibility of personalized CR, and training can be adjusted for the demands of activities of daily living. These four case reports describe recoveries from severe cases of IE, and an extreme recovery to a high level of fitness, demonstrating that CR is a safe component of IE recovery even in very sick patients and the potential effectiveness of personalized CR in helping patients reach their activity goals.

### 4.3 The Importance of Multi-Disciplinary Teams

**Fig. 2.**
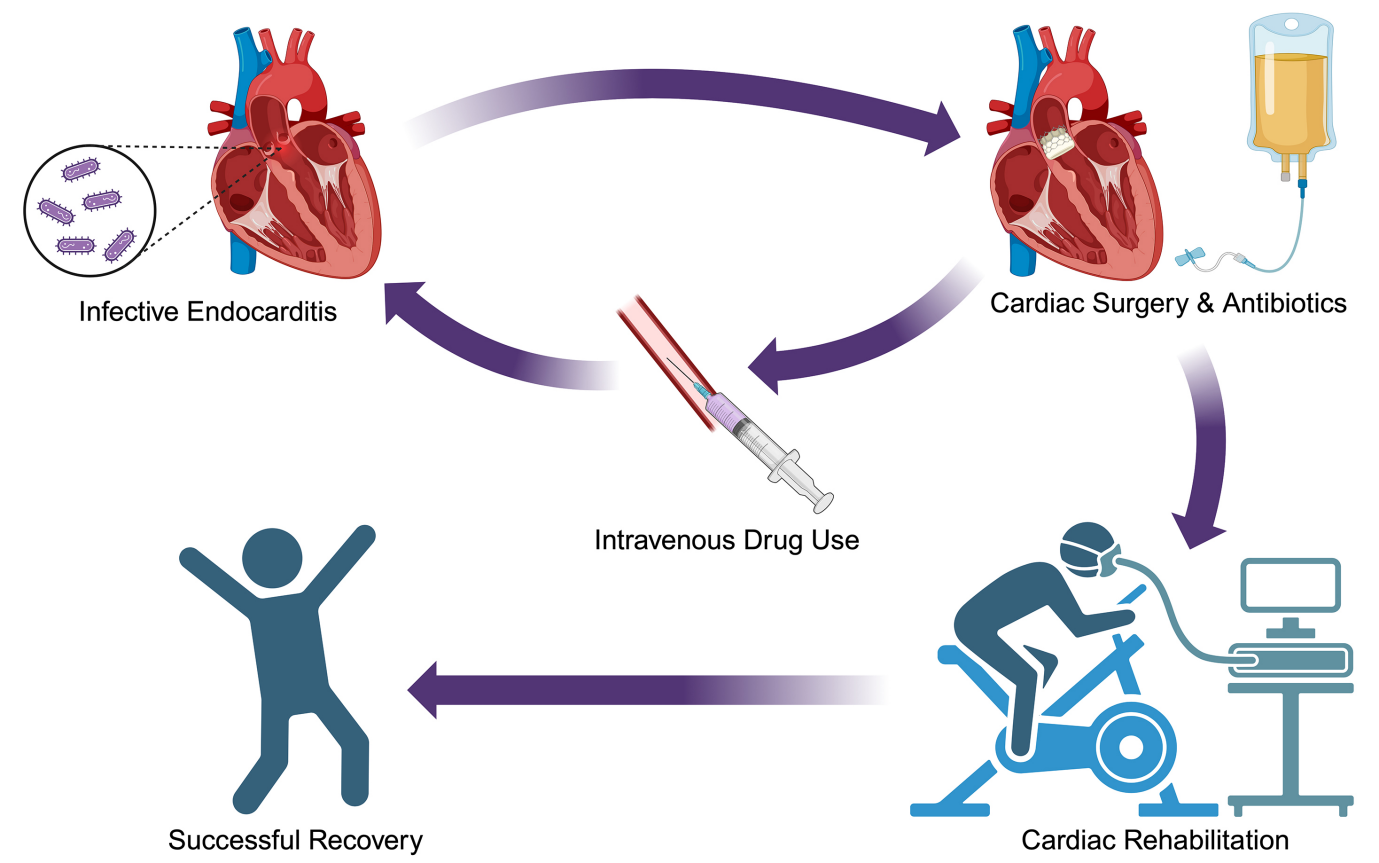
**Potential patient outcomes**. Graphical representation of two potential IE patient trajectories through CR or IE recurrence. IE, infective endocarditis; CR, cardiac rehabilitation. Figure created with BioRender.

It is important to consider how the recent shifts in the IE population may impact the most useful components of CR [2, 5]. With the increasing rates of IVDU-associated IE and the complex social needs of patients with a history of IVDU, integrating social workers into the multidisciplinary CR team could help with addiction recovery and decrease the risk of post-surgical complications and recurrent IE [5, 6]. Roberts *et al*. [6] recommend having social workers embedded within an addictions consult service to collaborate with hospital teams across multiple disciplines, coordinate patient care, provide case management and disposition planning, develop therapeutic rapport, and address stigma at both an individual and systemic level in order to better address the SDOH among hospitalized patients with a history of IVDU. These concepts could be incorporated into a CR program to specifically target the unique needs of IE patients and support their transition from the hospital back into the community (Fig. 2).

### 4.4 Cardiac Rehabilitation Following Heart Valve Surgery

Since valve replacement is the most common surgical treatment for IE [1, 2, 8], guidance surrounding the utility of CR after IE may be drawn from evidence supporting CR following valve surgery. In 2005, the ESC recommended that multidisciplinary CR should be offered to patients following valve surgery, as either an inpatient or outpatient program, depending on the local availability of CR programs and the pattern of the patient’s recovery [18]. They highlighted that submaximal exercise testing should guide individualized CR, as different patients can have different levels of exercise tolerance after heart valve surgery [18]. For example, exercise tolerance is typically lower following mitral valve replacement, especially in patients with persistent pulmonary hypertension, compared to exercise tolerance following aortic valve replacement [18]. They also discussed medical and surgical treatment of post-surgical IE of the prosthetic valve, but did not specifically discuss CR after IE treatment [18]. In 2017, Pollmann *et al*. [19] conducted a clinical trial examining CR after heart valve surgery. Patients underwent three months of individualized CR programs based on an initial exercise test. The results showed a 13–17% improvement in different measures of exercise capacity on the final exercise test, and showed reduced mortality for CR participants compared to the control group [19]. While these results are promising, the development of IE was only reported as a study endpoint for two patients undergoing CR and three control patients, so there is no specific evidence examining CR after IE treatment [19].

In 2021, Abraham *et al*. [20] published a meta-analysis of RCTs examining CR following heart valve surgery. They concluded that while the existing data show benefits for short-term exercise capacity and support the use of CR, the existing literature remains inadequate for definitive conclusions about the long-term impacts of CR on mortality, rehospitalizations, and quality of life [20]. Generally, CR programs are underused by many of the patient populations that could stand to benefit from such programs. One potential solution to increase the capacity, accessibility, and utilization of CR amongst many patient populations is to implement home-based CR [21]. Home-based CR has been shown to achieve similar patient improvements, and offering patients multiple options for accessing CR could reduce participation barriers, particularly for rural populations with limited access to traditional CR programs [21].

### 4.5 Limitations

The scope of this narrative review is limited primarily by the small number of studies examining CR in the context of IE. With only one RCT and four case reports, there are limited findings to discuss. While the RCT examined multiple outcomes of CR, the results were mostly inconclusive due to between-group differences at baseline, a lack of sufficient participants for statistical power, and poor participant adherence to the study protocol. While the four case reports offer some useful clinical perspectives and individualized CR protocols, they do not hold the same strength of evidence as larger trials. Additionally, there is an inherent risk of bias in the case reports towards supporting the treatment that was chosen by the authors, in this case, the use of CR. Overall, there is still a lack of evidence surrounding the effectiveness of CR following surgical treatment of IE, and further studies are warranted to examine both the physical and social impacts of CR for addressing the unique needs of the IE patient population. This narrative review is also limited by including only English-language articles available in the PubMed database. There are potentially additional studies available in other databases or published in non-English languages discussing CR following IE that were not included in the current review.

## 5. Conclusions

Considering the high morbidity of IE, as demonstrated by attributable DALYs [2], there is a clear need for improved recovery support. Based on the results of the CopenHeart_IE_ RCT, traditional standardized CR may be of value for improving patient recovery from IE [8]. Additionally, multiple case studies demonstrate the safety and feasibility of CR in IE recovery, and suggest that a more individualized approach may be beneficial for this unique patient population [14, 15, 16, 17]. While there is still a lack of definitive evidence, CR is potentially beneficial for patients’ physical health and emotional well-being and is very unlikely to be harmful. Further studies should be conducted to determine the measurable benefit and best practices for CR following IE.
